# High Oncological Efficacy of BCG Maintenance Therapy for Primary High-Grade T1 Urothelial Carcinoma of the Bladder

**DOI:** 10.3390/cancers18030532

**Published:** 2026-02-06

**Authors:** Takahide Noro, Naoto Kamiya, Naoki Ishitsuka, Rino Ikeda, Yuta Suzuki, Syota Iijima, Yuka Sugizaki, Takatoshi Somoto, Ryo Oka, Takanobu Utsumi, Takumi Endo, Nobuyuki Hiruta, Hiroyoshi Suzuki

**Affiliations:** 1 Department of Urology, Toho University Sakura Medical Center, 564-1 Shimoshizu, Sakura-shi 285-8741, Chiba, Japan; takahide.noro@med.toho-u.ac.jp (T.N.); naoki.ishitsuka@med.toho-u.ac.jp (N.I.); rino.ikeda@med.toho-u.ac.jp (R.I.); yuta.suzuki@med.toho-u.ac.jp (Y.S.); shouta.iijima@med.toho-u.ac.jp (S.I.); yuuka.kizuki@med.toho-u.ac.jp (Y.S.); takatoshi.soumoto@med.toho-u.ac.jp (T.S.); ryou.oka@med.toho-u.ac.jp (R.O.); takanobu.utsumi@med.toho-u.ac.jp (T.U.); takumi.endou@med.toho-u.ac.jp (T.E.); hiroyoshi.suzuki@med.toho-u.ac.jp (H.S.); 2 Department of Surgical Pathology, Toho University Sakura Medical Center, 564-1 Shimoshizu, Sakura-shi 285-8741, Chiba, Japan; nhr@med.toho-u.ac.jp

**Keywords:** bladder cancer, high-grade T1, intravesical BCG therapy, intravesical chemotherapy

## Abstract

**Background**: In high-risk non-muscle-invasive bladder cancer (NMIBC), adjuvant therapies, such as intravesical Bacillus Calmette–Guérin (BCG) instillation, are widely employed; however, BCG treatment poses challenges due to potential adverse effects and ongoing supply limitations. This study aimed to evaluate treatment patterns, therapeutic efficacy, incidence of adverse events, and clinical predictors of recurrence and progression in patients with high-grade pT1 urothelial carcinoma (HG-T1 UC) of the bladder. **Methods**: This retrospective cohort study included 204 patients diagnosed with HG-pT1 UC who underwent transurethral resection of bladder tumor (TURBT) at Toho University Sakura Medical Center between 2010 and 2021. Clinical data encompassing treatment modalities (BCG or intravesical chemotherapy), complications, and oncological outcomes were collected. Recurrence-Free Survival (RFS), Progression-Free Survival, and Cancer-Specific Survival were analyzed using Kaplan–Meier analyses and multivariate regression models. **Results**: Maintenance BCG therapy was significantly associated with prolonged RFS compared to other treatments, including among ‘very high-risk’ patients. However, 52.4% of patients receiving BCG maintenance experienced adverse events, with dose reductions required in 59% of cases. Notably, recurrence rates did not significantly differ based on dose reduction or the total number of BCG instillations. Tumor multiplicity emerged as an independent risk factor for recurrence. **Conclusions**: Although maintenance BCG therapy remains essential for managing HG-T1 UC, especially in high-risk patients, treatment should be individualized due to concerns about tolerability and availability. The study results support the importance of personalized strategies based on risk stratification as outlined in clinical guidelines for preventing recurrence in NMIBC.

## Simple Summary

High-grade pT1 urothelial carcinoma (HG-T1 UC) is a non-muscle-invasive bladder cancer with a high risk of recurrence and progression. We retrospectively analyzed 204 patients treated at Toho University Sakura Medical Center to evaluate real-world outcomes of intravesical therapies, mainly Bacillus Calmette–Guérin (BCG) and intravesical chemotherapy. Maintenance BCG therapy significantly prolonged recurrence-free survival compared with other treatments, even in very high-risk cases. However, over half of the patients experienced adverse events, and dose reductions were required in approximately 60% of them. These reductions did not increase recurrence rates. Tumor multiplicity was identified as an independent risk factor for recurrence. This study clarifies the efficacy and challenges of BCG maintenance therapy. For the primary treatment of HG-T1 UC of the bladder, intravesical BCG maintenance therapy should be implemented, incorporating flexible dose reductions and treatment interruptions to effectively manage adverse events.

## 1. Introduction

Bladder cancer is one of the most common malignancies in the field of urology, with an estimated 610,000 new cases diagnosed worldwide each year, and approximately 220,000 deaths reported from any cause [1,2]. In Japan as well, bladder cancer has shown a rising trend, with approximately 25,000 new cases diagnosed and around 10,000 deaths reported annually [3]. Approximately 75% of these cases are identified as non-muscle invasive bladder cancer (NMIBC), for which transurethral resection of bladder tumor (TURBT) is considered as the initial therapeutic intervention [4,5]. Within the spectrum of NMIBC, high-grade pT1 urothelial carcinoma (HG-T1 UC) is characterized by submucosal invasion without muscular involvement, and is associated with significantly higher rates of recurrence and progression compared to other NMIBC subtypes, such as Ta and low-grade tumors [6,7]. Given the higher risk of recurrence and progression associated with these high-risk NMIBCs, postoperative adjuvant therapy is strongly recommended, since TURBT alone is frequently insufficient. Among the available modalities, intravesical Bacillus Calmette-Guérin (BCG) instillation therapy has been extensively utilized since the 1970s, and is currently regarded as the standard adjuvant treatment for high-risk NMIBC. Notably, numerous national and international clinical trials have demonstrated that maintenance BCG therapy, administered over a period ranging from several months to years, significantly reduces recurrence rates and disease progression [8,9,10].

Nonetheless, intravesical BCG therapy is accompanied by considerable clinical challenges. These include a high incidence of adverse effects, encompassing not only local symptoms such as dysuria, urinary frequency, and bladder discomfort, but also rare, albeit serious, systemic complications, including urinary tract infections (UTIs), febrile episodes, and sepsis [11]. In addition, the global shortage of BCG supply has heightened the need for alternative therapeutic approaches and personalized treatment strategies [12,13]. In patients for whom BCG therapy is contraindicated or poorly tolerated, intravesical chemotherapy with anticancer agents might serve as an alternative. However, several meta-analyses and randomized controlled trials (RCTs) have consistently shown that intravesical chemotherapy is markedly less effective than BCG in preventing tumor recurrence [14], particularly in high-risk populations such as those with HG-T1 UC, in whom therapeutic efficacy might be suboptimal.

Moreover, HG-T1 UC represents a biologically heterogeneous entity, and accurately predicting the risk of recurrence and progression remains challenging. Various clinicopathological parameters—including age, concomitant carcinoma in situ (CIS), tumor multiplicity, tumor size, lymphovascular invasion (LVI), and presence of residual tumor at re-TURBT—have been implicated as influencing disease course, although a consensus on their practical utility in guiding treatment decisions has not been firmly established [4,15,16]. Recent studies underscore the clinical utility of advanced diagnostic and analytical frameworks in optimizing risk stratification for NMIBC; however, these techniques have yet to be widely implemented in routine clinical practice [17,18].

Despite robust evidence from randomized controlled trials supporting maintenance BCG therapy for high-risk NMIBC, a substantial gap remains between guideline recommendations and real-world clinical practice. Specifically, in elderly patients and those with comorbidities such as diabetes or collagen diseases, treatment-limiting toxicities, dose reductions, interruptions, or discontinuations are frequently encountered, driven by adverse events (AEs) or global BCG supply constraints. Consequently, the oncological impact of non-standardized or incomplete BCG maintenance schedules in routine clinical settings has not been fully elucidated. The objective of this study was to evaluate real-world treatment patterns, oncological outcomes, and AEs in patients with primary HG-T1 UC of the bladder treated with intravesical therapy. We also investigated the clinical effectiveness of BCG maintenance therapy under various treatment conditions, including dose modification and treatment interruption.

Furthermore, although recent investigations have indicated that photodynamic diagnosis (PDD) or narrow band imaging (NBI) during initial TURBT might enhance tumor visualization and reduce recurrence rates [19,20], this study excluded cases in which PDD and NBI were employed, in order to isolate the outcomes associated with conventional white-light TURBT, thereby ensuring that the findings only reflect standard TURBT protocols.

## 2. Materials and Methods

This retrospective cohort study was conducted at Toho University Sakura Medical Center, a prominent regional institution recognized for its expertise in the diagnosis and treatment of bladder cancer, in accordance with the principles of the Declaration of Helsinki and after receiving approval from the Ethics Committee of Toho University Sakura Medical Center (Approval no.: S23064; Approval date: 5 April 2024). The study protocol was publicly disclosed on the institutional website, allowing patients the opportunity to opt out, if desired. All patients who underwent TURBT for bladder cancer between April 2010 and December 2021 were screened for eligibility. The study period was set from 2010 to 2021 to ensure an adequate follow-up duration for evaluating recurrence and survival outcomes following intravesical therapy. This timeframe aligns with the period when BCG maintenance therapy was consistently recommended for high-risk NMIBC by major clinical guidelines. Among them, 204 patients with complete clinical data and an initial pathological diagnosis of HG-T1 UC were included in the final analysis. Patients with missing or incomplete clinical records were excluded from the study population during the initial screening process. Cases involving PDD-assisted TURBT were excluded to ensure that outcomes were based solely on conventional white-light TURBT.

Clinical data were meticulously extracted from the hospital’s electronic medical records, including demographic characteristics, clinical profiles, histopathological findings, performance of a second TURBT, details of adjuvant therapies administered, treatment-related complications, and clinical outcomes. For BCG intravesical therapy, the Tokyo 172 strain (Japan BCG Laboratory, Tokyo, Japan) was utilized. Intravesical chemotherapy consisted of pirarubicin (30 mg) (Meiji Seika Pharma Co., Ltd., Tokyo, Japan) administered once weekly for a total of eight instillations. Patients who received BCG therapy were stratified based on the completion of at least five of six induction instillations and at least two of three maintenance instillations, resulting in a cumulative total of seven or more BCG instillations. For intravesical chemotherapy, 30 mg of pirarubicin was administered once weekly for a total of eight doses. No maintenance chemotherapy was provided in this protocol [21]. Treatment-related AEs were graded according to the Common Terminology Criteria for Adverse Events version 5.0.

Four primary clinical endpoints were evaluated: progression-free survival (PFS), recurrence-free survival (RFS), cancer-specific survival (CSS), and overall survival (OS). PFS was defined as the interval from the initial TURBT to the development of muscle-invasive bladder cancer, RFS as the time from TURBT to intravesical recurrence, CSS as the duration from TURBT to death attributable to bladder cancer, and OS as the duration from TURBT to death from any cause.

Statistical analyses were performed using JMP Pro version 17.0.0. Appropriate statistical methods were employed, including Student’s *t*-test, Fisher’s exact test, log-rank test, Kaplan–Meier analyses, and logistic regression modeling. A two-sided *p*-value of less than 0.05 was considered statistically significant.

## 3. Results

Table 1 summarizes the characteristics of the study population. A total of 204 patients diagnosed with HG-T1 UC who underwent TURBT were included in this study.

The median follow-up period after TURBT was 63.3 months (range: 0.5–121.6 months). Median age at TURBT was 74 years (range: 40–93 years), with 48 female patients (23.5%). A history of smoking was present in 68.6% of patients, and 40.7% had positive urinary cytology results prior to treatment. Among the patients, 111(54.4%) had multiple tumors, 54 (26.4%) had Grade 3 (G3) tumors, 64 (31.5%) were positive for CIS, 25 (12.6%) were positive for LVI, and 13 (6.3%) had urothelial carcinoma (UC) of variant histology. The breakdown of UC variants is as follows: glandular differentiation in six cases, squamous differentiation in five cases, sarcomatoid variant in one case, and micropapillary variant in one case.

A second transurethral resection of bladder cancer (second TUR) was performed in 102 patients (50.0%), and residual tumor was detected in 38 cases (37.6%). After the initial or second TUR, treatments included maintenance BCG therapy in 61 patients (29.9%), BCG induction therapy in 46 (22.5%), intravesical chemotherapy in 24 (11.7%), immediate radical cystectomy in 13 (6.3%), and observation alone in 60 patients (29.4%) (Supplementary Figure S1).

There was no significant difference in age between the observation and treatment groups (median age: observation 76 years [interquartile range (IQR) 12.0] vs. treatment 74 years [IQR 11.0], *p* = 0.09). According to the Japanese Urological Association (JUA) guidelines, 166 patients (81.3%) were classified as very high-risk, whereas under the European Association of Urology (EAU) guidelines, 50 patients (24.5%) fell into this category [5,20].

Intravesical recurrence occurred in 63 cases (30.8%), with a median RFS period of 11.9 months. The treatment sequence of the patients in this study is shown in Supplementary Figure S1. During the observation period, 24 patients (11.8%) underwent radical cystectomy. The treatment course prior to cystectomy included: immediate cystectomy in 13 cases (54.2%), BCG induction in seven cases (29.2%), observation in two cases (8.3%), BCG maintenance in one case (4.2%), and intravesical chemotherapy in one case (4.2%). The median time from TURBT to cystectomy was 4.7 months. Pathological staging of cystectomy specimens revealed pT < 2 in 17 cases (70.8%) and pT ≥ 2 in seven cases (29.2%).

**Figure 1:**
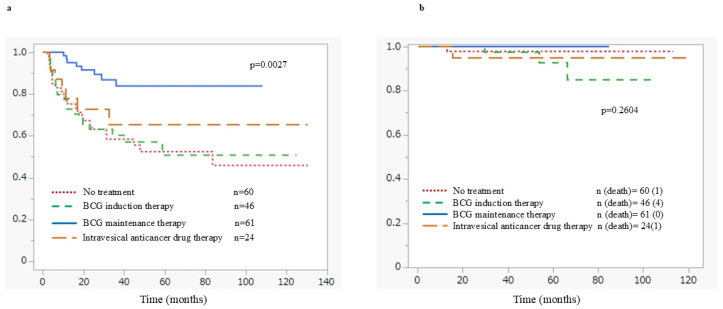
Survival with each additional treatment in pT1 high-grade bladder cancer patients; (**a**) Recurrence-free survival; (**b**) Cancer-specific survival.

Recurrence rates and time to recurrence by treatment modality are shown in Supplementary Table S1. Intravesical recurrence occurred in 13.1% of patients receiving BCG maintenance therapy, which was lower than the rates observed in the BCG induction therapy (41.3%), intravesical chemotherapy (29.2%), and no-treatment groups (40.0%). Moreover, the 5-year intravesical non-recurrence rate was highest in the BCG maintenance therapy group (86.9%), compared with 58.7% in the BCG induction therapy group, 70.8% in the intravesical chemotherapy group, and 61.6% in the no-treatment group. No significant difference in RFS was observed between the chemotherapy group and BCG induction group. However, the BCG maintenance group showed significantly longer RFS than the BCG induction group (*p* < 0.05) (Figure 1a). Even among very high-risk patients, RFS was significantly better in the BCG maintenance group compared to other treatments (*p* < 0.05). Progression to muscle-invasive disease was observed in 11 patients (5.4%), with a median PFS of 27.4 months. During the observation period, 36 patients (17.6%) died, with a median OS of 34.0 months. The remaining causes of death were senility in ten cases, other malignant neoplasms in nine, cardiovascular diseases in four, infectious diseases in four, and suicide in one case. Cancer-specific mortality occurred in eight patients (3.9%), with a median time to CSS of 22.6 months. There were no significant differences in CSS among the treatments. However, there were no patients with cancer-related death in the BCG maintenance therapy group. On the other hand, three patients in the BCG induction therapy group died of bladder cancer (Figure 1b).

Regarding AEs, none occurred in the intravesical chemotherapy group. In the BCG maintenance group, 32 patients (52.4%) experienced AEs. Most were lower urinary tract symptoms, such as dysuria and frequent urination. Serious complications included urinary tract tuberculosis in two cases, Reiter’s syndrome in one case, and Pott’s disease (spinal tuberculosis) in one case. All four severe complications were successfully treated with oral anti-tuberculosis medication and corticosteroids.

In the BCG maintenance therapy group, the median cumulative number of instillations was 15, with dose reductions required in 36 patients (59.0%). Furthermore, 32 patients (52.5%) discontinued BCG therapy due to treatment-related AEs. Notably, no statistically significant differences in RFS were observed based on dose reduction, total number of instillations, or the occurrence of AEs (Supplementary Figure S2a–c). No cancer-specific deaths were observed among patients who received BCG maintenance therapy. Progression to muscle-invasive disease occurred in one patient in the continuation group (*n* = 29), and PFS did not differ significantly between the discontinuation and continuation groups. Among the 32 patients who discontinued BCG maintenance therapy, all were managed with observation alone. Intravesical recurrence was identified in three patients and was managed with TURBT, followed by BCG re-challenge in one patient, intravesical pirarubicin therapy in one patient, and observation alone in one patient. No recurrence was observed in the remaining 29 patients, who continue under observation.

The results of Cox proportional hazards model analyses for endpoints including intravesical recurrence, progression to muscle-invasive disease, cancer-specific mortality, and OS are shown in Table 2.

**Figure 2:**
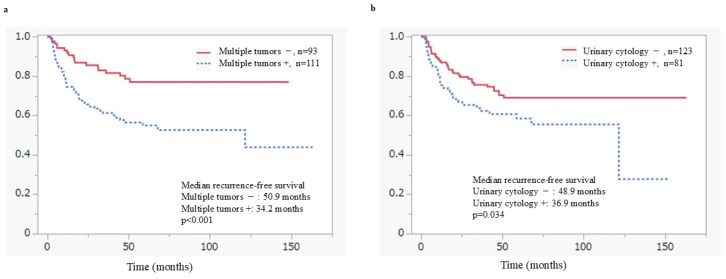
Recurrence-free survival according to risk factors in T1 high-grade bladder cancer patients; (**a**) Recurrence-free survival by tumor multiplicity; (**b**) Recurrence-free survival by urinary cytology at initial TURBT.

**Figure 3:**
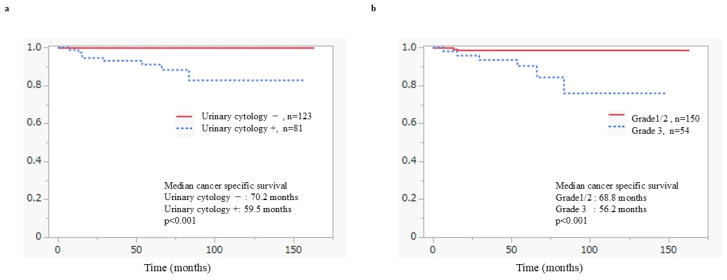
Cancer-specific survival according to risk factors in T1 high-grade bladder cancer patients; (**a**) Cancer-specific survival by urinary cytology at initial TURBT; (**b**) Cancer-specific survival by tumor grade status.

In univariate Cox proportional hazards analyses and Kaplan–Meier analyses, positive urinary cytology, tumor multiplicity, and the absence of maintenance BCG therapy were significantly associated with an increased risk of intravesical recurrence. In multivariate analyses, tumor multiplicity (RFS: hazard ratio [HR] 2.45, 95% confidence interval [CI] 1.43–4.38, *p* = 0.001) and the absence of maintenance BCG therapy (RFS: HR 0.36, 95% CI 0.18–0.66, *p* = 0.001) remained independently associated with a higher risk of recurrence. In addition, positive urinary cytology at the time of initial TURBT was significantly associated with inferior progression-free survival (PFS: HR 5.42, 95% CI 1.29–36.6, *p* = 0.036) as well as poorer cancer-specific survival. High tumor grade also contributed to an increased risk of cancer-specific and overall mortality (CSS: HR 5.53, 95% CI 1.26–38.1, *p* = 0.037) (Figure 2b and Figure 3b). Similarly, the presence of carcinoma in situ (CIS) was identified as a predictor of unfavorable prognosis and showed a significant independent association with overall survival in multivariate analysis (OS: HR 2.41, 95% CI 1.16–4.96, *p* = 0.016). Other prognostic factors, including age ≥ 75 years and the absence of a second TUR, were significantly associated with overall survival in univariate analysis, and age ≥ 75 years remained an independent predictor of poorer overall survival in multivariate analysis (OS: HR 2.60, 95% CI 1.25–5.76, *p* = 0.012).

Figure 2 shows RFS in patients with HG-T1 UC in the bladder, stratified by risk factors using the Kaplan–Meier analyses. Both tumor multiplicity (*p* < 0.001) (Figure 2a) and positive urinary cytology at the time of initial TURBT (*p* = 0.034) (Figure 2b) were identified as significant predictors of recurrence. Figure 3 shows CSS according to risk factors in patients with HG-T1 UC in the bladder, estimated using the Kaplan–Meier analyses. Positive urinary cytology at the time of initial TURBT (*p* < 0.001) and G3 tumor grade (*p* < 0.001) were identified as significant poor prognostic factors.

## 4. Discussion

This retrospective study involving 204 patients diagnosed with HG-T1 UC in the bladder investigated intravesical recurrence rates and associated predictive factors across different treatment modalities. HG-T1 UC exhibits a notably high risk of recurrence and progression among NMIBCs, necessitating careful consideration of patient demographics, pathological features, and treatment responses when determining therapeutic strategies [21]. Moreover, given the pronounced heterogeneity of these tumors, risk stratification and personalized treatment approaches are critically important [22].

In this cohort, the group receiving BCG maintenance therapy exhibited a significantly lower rate of intravesical recurrence (*p* < 0.05) and prolonged RFS compared with other treatment groups. These results align with those of previous large-scale randomized controlled trials and meta-analyses that have established BCG maintenance as one of the most effective strategies for reducing recurrence and progression in NMIBC [21]. For instance, a meta-analysis by Sylvester et al. showed that one year of maintenance BCG therapy following induction reduced recurrence by approximately 32%, particularly in patients with CIS and those at high risk for recurrence [23]. On the other hand, the lack of significant differences in PFS and CSS is likely attributable to the limited number of progression and cancer-related death events, resulting in insufficient statistical power. In addition, the performance of RC in selected cases may have influenced these survival outcomes.

In the present study, 81.3% of patients were classified as very high-risk according to JUA guidelines, and 24.5% according to EAU guidelines [5,24], indicating that the study population mirrors a real-world cohort with considerable clinical risk. Classification as very high-risk includes adverse prognostic features such as presence of CIS, LVI, BCG-refractory status, variant histologies, and frequent tumor recurrence. Despite an ongoing debate regarding the optimal management of this population, our findings highlight the efficacy of maintenance BCG therapy even in very high-risk patients (*p* < 0.05). BCG is recognized as enhancing both innate and adaptive immune responses within the bladder, fostering a microenvironment conducive to tumor eradication [25]. These outcomes reinforce the therapeutic potential of BCG in managing aggressive or multifocal lesions. In particular, maintenance BCG therapy is strongly recommended for patients classified as very-high-risk under JUA criteria, given its clear benefit in prolonging RFS. Previous studies involving NMIBC with variant histologies have shown that both BCG maintenance and radical cystectomy improve OS as compared to observation alone, further supporting BCG as a viable therapeutic option [26]. However, no significant difference in PFS was observed with maintenance BCG therapy, even on univariate analysis. Therefore, careful radiologic surveillance with computed tomography and magnetic resonance imaging may be warranted to detect tumor progression in patients with HG-T1 UC.

Observation alone was followed in 29.4% of cases in this study. The median age of such patients was 76 years, with no statistically significant difference in age between the observation and treatment groups (*p* = 0.09). This suggests that other factors, such as physician discretion, patient preferences, and tumor characteristics might have influenced treatment selection. International studies have reported wide variability in adherence to NMIBC treatment guidelines across countries, particularly in risk-adapted management and surveillance protocols [27,28]. In Japan, regional and institutional variations in treatment strategies may exist, and these findings underscore the importance of adherence to clinical guidelines in patients who are eligible for maintenance BCG therapy.

The cohort receiving intravesical chemotherapy (e.g., epirubicin or mitomycin C) in this study did not show a significant RFS benefit compared with observation alone (*p* = 0.58), suggesting its limited efficacy in this high-risk setting. The fact that all patients in this study had HG-T1 disease suggests that monotherapy with intravesical chemotherapy might be inadequate in this cohort. Consistent with the findings by Aghamir et al., our results further support maintenance BCG therapy as the preferred treatment for high-risk NMIBC [29]. In addition to therapeutic efficacy, the incidence and severity of adverse effects of the various treatment modalities are critical considerations in treatment selection. In the maintenance BCG cohort, 52.4% of patients experienced treatment-related complications, predominantly as localized symptoms such as dysuria, urinary frequency, and hematuria. However, serious systemic AEs—including urinary tract infections, Reiter’s syndrome, and vertebral osteomyelitis—also occurred, which were attributable to the live-attenuated nature of the BCG strain, warranting clinical vigilance [30]. However, within the BCG maintenance group, no statistically significant differences in RFS were observed regardless of the occurrence of adverse events, dose reduction, or the total number of instillations (Supplementary Figure S2a–c). These findings suggest that prioritizing the continuation of maintenance therapy, even with dose adjustments to manage toxicity, is more critical for recurrence prevention than limiting treatment to induction therapy alone.

In multivariate Cox proportional hazards analysis, positive urinary cytology, tumor multiplicity, and failure to complete maintenance BCG therapy were identified as independent predictors of recurrence. In particular, positive urinary cytology appears to reflect a biologically aggressive tumor phenotype. In patients with positive cytology and BCG-refractory disease, early radical cystectomy should be considered. Tumor multiplicity was identified as an independent predictor of intravesical recurrence, consistent with established prognostic models such as the EORTC risk tables and the concept of field cancerization [7,31].

Residual tumor was detected at the second TUR in 38 patients (37.6%), suggesting possible initial understaging and highlighting the importance of complete resection of HG-T1 UC. High-quality initial TUR is reportedly associated with reduced recurrence, underscoring the importance of surgical precision in tumor control [32]. Since a second TUR enables accurate pathological staging and informed treatment planning, current guidelines advocate for its routine use in HG-T1 cases [5,33]. By improving staging accuracy, the second TUR enhances RFS and optimizes BCG efficacy [34,35]. Nevertheless, only 50% of patients (n = 102) in this study underwent a second TUR. Patients who did not undergo a second TUR were significantly older (78 years [IQR 11.3] vs. 72 years [IQR 11.0], *p* < 0.01), suggesting that advanced age and comorbidities may have constituted barriers to invasive treatment, thereby potentially contributing to inferior overall survival (*p* < 0.05). In addition, the association between the presence of CIS and poorer overall survival likely reflects the inherent difficulty in controlling occult or microscopically progressive disease. Furthermore, the finding that omission of a second TUR was associated with reduced overall survival underscores the critical importance of accurate assessment of residual disease and adequate local tumor control.

In summary, our findings reinforce the therapeutic value of BCG maintenance therapy in managing HG-T1 UC in the bladder and emphasize the need for individualized treatment strategies, particularly in older adults and patients with CIS. Our findings also suggest that the anti-tumor efficacy of BCG induction therapy is limited. Conversely, maintenance BCG therapy demonstrated high clinical effectiveness, even in cases of incomplete protocols or dose reductions. These results could be explained by the immunological mechanisms of BCG, which suggest that effective antitumor immunity is established during the induction and initial maintenance stages. Since intravesical BCG has been shown to induce rapid innate and adaptive immune responses, the quality of initial immune activation may be more decisive for oncological outcomes than the total cumulative dose [36,37]. While concerns regarding AEs and stable supply persist, at present, it is important to administer BCG maintenance therapy whenever feasible for patients with HG-T1 UC of the bladder, even if strict adherence to the standard protocol is difficult. For BCG-refractory disease, early RC should be considered rather than prolonged bladder-sparing attempts. Given the current instability in global BCG supply, we believe that priority should be given to patients with HG-T1 UC in the bladder who have recurrence or poor prognostic factors, such as positive urine cytology, multifocal tumors, LVI positivity, or the presence of variant histology. Furthermore, there is an urgent need to develop alternative therapeutic approaches to BCG. Currently, immune checkpoint inhibitors are under active investigation as potential therapeutic options in BCG-refractory NMIBCs and might become integral to future treatment paradigms [38,39].

This study has several limitations inherent to its retrospective, single-center design. Treatment allocation was not randomized and was influenced by physician discretion, patient preference, age, and comorbidities, which may have introduced selection bias. Although these factors limit the generalizability of our findings, the inclusion of a relatively large high-risk cohort provides valuable insights into the real-world management of HG-T1 UC of the bladder. Future multi-institutional studies with larger patient populations and longer follow-up periods are needed to validate these results and to further refine evidence-based treatment strategies for patients with HG-T1 UC of the bladder. Therefore, we plan to conduct a multi-institutional study in the future.

## 5. Conclusions

This retrospective analysis of 204 patients with high-grade T1 UC of the bladder identified tumor multiplicity and positive urinary cytology as key prognostic factors associated with recurrence and unfavorable oncological outcomes. Intravesical BCG maintenance therapy significantly prolonged RFS compared with BCG induction therapy and intravesical chemotherapy. For the primary treatment of HG-T1 UC of bladder, intravesical BCG maintenance therapy should be implemented, incorporating flexible dose reductions and treatment interruptions to effectively manage AEs. However, no statistically significant association was observed with disease progression or CSS, indicating that the interpretation of its therapeutic benefit should remain cautious. In the face of ongoing BCG shortages and the emergence of novel therapeutic options, such as immune checkpoint inhibitors, future prospective multi-center studies are essential to validate these findings and delineate alternative strategies for patients with BCG-unresponsive disease.

## Figures and Tables

**Table 1:** Characteristics of the study population.

Variables	Total (*n* = 204)
Median follow-up duration after TURBT (months, range)	63.3 (0.5–121.6)
Age at TURBT, median (range)	74 (40–93)
Females, *n* (%)	48 (23.5)
Positive smoking history, *n* (%)	140 (68.6)
Positive urinary cytology, *n* (%)	83 (40.7)
Tumor size ≥ 3 cm, *n* (%)	55 (27.0)
Multiple tumors, *n* (%)	111 (54.4)
Papillary type cancer, *n* (%)	188 (92.1)
Presence of G3, *n* (%)	54 (26.4)
CIS positivity, *n* (%)	64 (31.5)
LVI positivity, *n* (%)	25 (12.6)
Variant histology present, *n* (%)	13 (6.8)
Second TUR performed, *n* (%)	102 (50.0)
Cases with residual tumor at second TUR, *n* (%)	38 (37.6)
Treatment after TURBT	
BCG maintenance therapy, *n* (%)	61 (29.4)
BCG induction therapy, *n* (%)	46 (22.5)
Intravesical chemotherapy, *n* (%)	24 (11.7)
Cystectomy, *n* (%)	13 (6.3)
No treatment, (%)	60 (29.4)
JUA VHR, *n* (%)	166 (81.3)
EAU VHR, *n* (%)	50 (24.5)
Intravesical recurrence, *n* (%)	63 (30.8)
Median time to intravesical recurrence, months (range)	11.9 (2.8–83.5)
Cystectomy during the observation period, *n* (%)	24 (11.8)
Treatment up to cystectomy (*n* = 24)	
BCG maintenance therapy, *n* (%)	1 (4.2)
BCG induction therapy, *n* (%)	7 (29.2)
Intravesical chemotherapy, *n* (%)	1 (4.2)
Immediate cystectomy, *n* (%)	13 (54.2)
No treatment, *n* (%)	2 (8.3)

TURBT: transurethral resection of bladder tumor, CIS: carcinoma in situ, LVI: lymphovascular invasion, BCG: Bacillus Calmette-Guérin, JUA: Japanese Urological Association, VHR: very high risk, EAU: European Association of Urology.

**Table 2:** Estimates of recurrence-free survival, progression-free survival, cancer-specific survival, and overall survival using Cox proportional hazards models among patients diagnosed with pT1 high-grade bladder cancer.

	RFS		PFS		CSS		OS	
	Univariate	Multivariate	Univariate	Multivariate	Univariate	Multivariate	Univariate	Multivariate
	*p*-Value	HR (95% CI)	*p*-Value	HR (95% CI)	*p*-value	HR (95% CI)	*p*-Value	HR (95% CI)
		*p*-Value		*p*-Value		*p*-Value		*p*-Value
Age in years (<75/75)	0.928		0.9845		0.3052		0.0028	2.6092
								1.2534–5.7616
								0.0129
Male/Female	0.5374		0.7165		0.4966		0.0892	
Urinary cytology (−/+)	0.0359	1.5047	0.0085	5.4239	0.0050	1.7655	0.1393	
		0.9137–2.4766		1.2983–36.621		0.8932–1.9126		
		0.1066		0.0360		0.0029		
Grade (G1, 2/G3)	0.5774		0.3181		0.0040	5.5392	0.0168	1.8565
						1.2610–38.109		0.8930–3.7443
						0.0374		0.0881
Tumor size (<3/3 cm)	0.8339		0.9055		0.9174		0.6451	
Tumor number (single/multiple)	0.0008	2.4565	0.5365		0.6158		0.9164	
		1.4359–4.3840						
		0.0015						
Variant histology (−/+)	0.4472		0.9990		0.9987		0.9997	
CIS (−/+)	0.7498		0.5421		0.3980		0.0055	2.4613
								1.1608–4.9669
								0.0165
LVI (−/+)	0.1158		0.0817	2.8846	0.3527		0.0889	
				0.6032–11.071				
				0.1374				
second TUR (−/+)	0.4394		0.0785	0.3182	0.0959	0.3972	0.0194	0.5280
				0.0469–1.3402		0.0575–1.7529		0.2475–1.0738
				0.1572		0.2630		0.0852
BCG maintenance therapy (−/+)	0.0038	0.3673	0.1311		0.0571	1.3130	0.0870	
		0.1869–0.6647				0.9828–1.9532		
		0.0018				0.0588		

## Data Availability

All relevant data supporting the findings of this study are included within the article and its Supplementary Materials. Due to patient privacy and institutional regulations, individual-level clinical data are not publicly available. Additional de-identified data may be obtained from the corresponding author upon reasonable request and with permission of the Toho University Sakura Medical Center Personal Information Protection Commission.

## Supplementary Materials

The following supporting information can be downloaded at https://www.mdpi.com/article/10.3390/cancers18030532/s1, Supplementary Table S1. Recurrence rate and time to recurrence after each additional treatment; Supplementary Figure S1. Flowchart of the study population and treatment sequence; Supplementary Figure S2. (a) Recurrence-free survival by number of BCG instillations in the BCG maintenance therapy group; (b) Recurrence-free survival with or without BCG dose reduction in the BCG maintenance therapy group; (c) Recurrence-free survival according to BCG discontinuation due to treatment-related adverse events in the BCG maintenance therapy group.
