# Supplementary material for: High Oncological Efficacy of BCG Maintenance Therapy for Primary High-Grade T1 Urothelial Carcinoma of the Bladder
Source: Cancers (Basel). 2026 Feb 6;18(3):532. doi: 10.3390/cancers18030532 (PMC12896884; doi:10.3390/cancers18030532)

Supplementary Figure S1

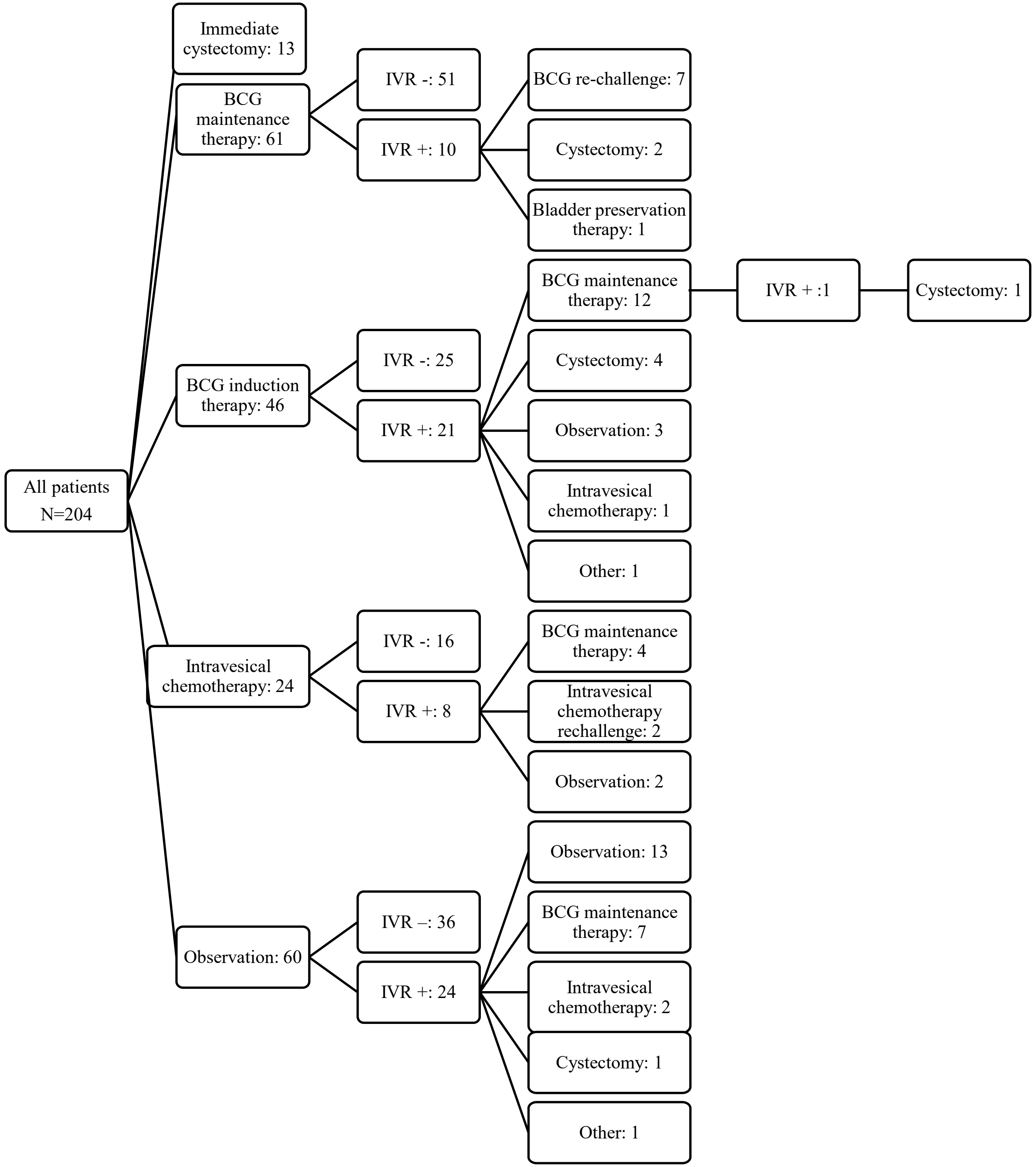

Supplementary Figure S2a

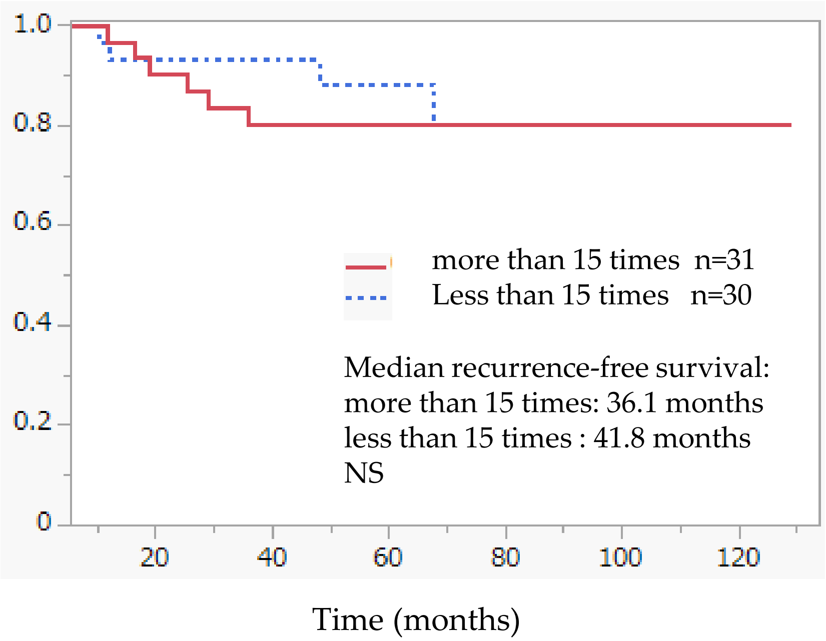

Supplementary Figure S2b

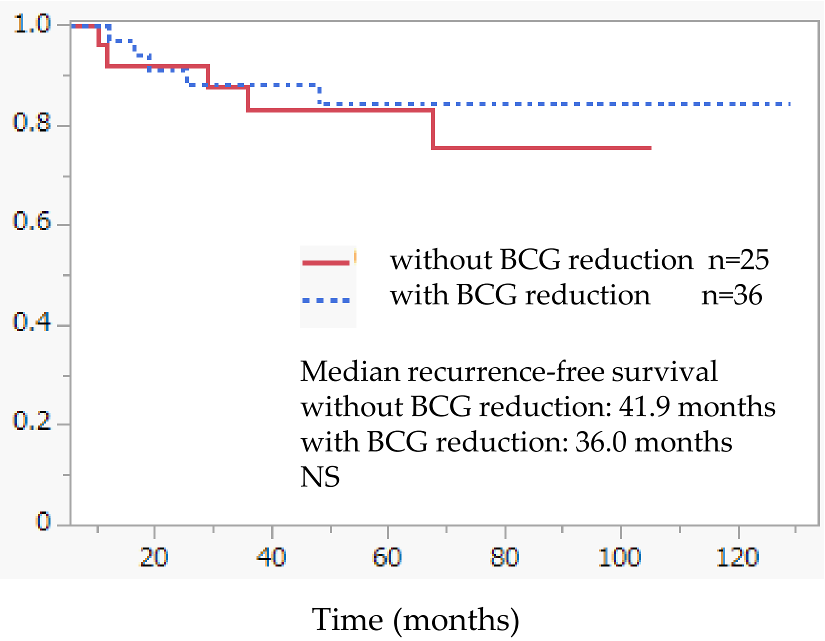

Supplementary Figure S2c

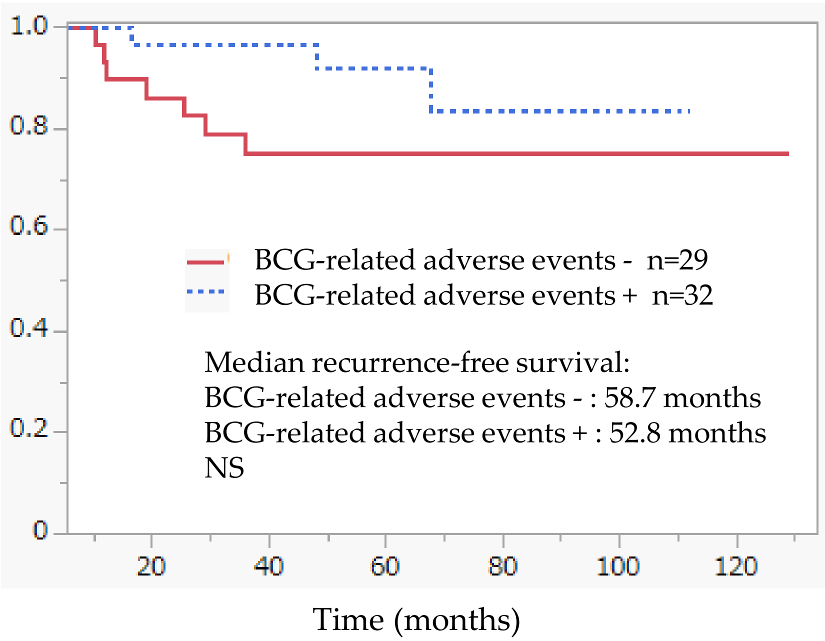

Supplement: Supplementary file 1 [file cancers-18-00532-s001.zip › Supplementary Figures.pdf]
