# Supplementary material for: High Oncological Efficacy of BCG Maintenance Therapy for Primary High-Grade T1 Urothelial Carcinoma of the Bladder
Source: Cancers (Basel). 2026 Feb 6;18(3):532. doi: 10.3390/cancers18030532 (PMC12896884; doi:10.3390/cancers18030532)
Supplement: Supplementary file 1 [file cancers-18-00532-s001.zip › Supplementary Table S1.pdf]

Supplementary File

Supplementary Table S1. Recurrence rate and time to recurrence after each additional treatment

	BCG maintenance therapy	BCG induction therapy	Intravesical chemotherapy	No treatment
n	61	46	24	60
Intravesical recurrence, n (%)	8 (13.1)	19 (41.3)	7 (29.2)	24 (40.0)
Median time to intravesical recurrence, months (range)	17.8 (10.2-36.1)	10.4 (2.8-58.8)	9.1 (2.9-32.5)	11.4 (2.4-83.5)
2-year non-recurrence rate (%)	91.8	65.2	75.0	68.3
5-year non-recurrence rate (%)	86.9	58.7	70.8	61.6

BCG: Bacillus Calmette-Guérin
